# The Developmental Origins of Voice Processing in the Human Brain

**DOI:** 10.1016/j.neuron.2010.03.001

**Published:** 2010-03-25

**Authors:** Tobias Grossmann, Regine Oberecker, Stefan Paul Koch, Angela D. Friederici

**Affiliations:** 1 Centre for Brain and Cognitive Development, Birkbeck, University of London, Malet Street, London WC1E 7HX, UK; 2 Department of Neuropsychology, Max Planck Institute for Human Cognitive and Brain Sciences, Stephanstrasse 1a, 04103 Leipzig, Germany; 3 Berlin Neuroimaging Centre, Department of Neurology, Charite Universitätsmedizin, Luisenstrasse 56, 10099 Berlin, Germany

**Keywords:** sysneuro

## Abstract

In human adults, voices are processed in specialized brain regions in superior temporal cortices. We examined the development of this cortical organization during infancy by using near-infrared spectroscopy. In experiment 1, 7-month-olds but not 4-month-olds showed increased responses in left and right superior temporal cortex to the human voice when compared to nonvocal sounds, suggesting that voice-sensitive brain systems emerge between 4 and 7 months of age. In experiment 2, 7-month-old infants listened to words spoken with neutral, happy, or angry prosody. Hearing emotional prosody resulted in increased responses in a voice-sensitive region in the right hemisphere. Moreover, a region in right inferior frontal cortex taken to serve evaluative functions in the adult brain showed particular sensitivity to happy prosody. The pattern of findings suggests that temporal regions specialize in processing voices very early in development and that, already in infancy, emotions differentially modulate voice processing in the right hemisphere.

## Highlights

► Temporal cortex specializes in processing human voices during infancy ► Emotion specifically enhances voice processing in the right hemisphere in infants ► Deeper evaluation of happy speech in infants' right inferior frontal cortex

## Introduction

The human voice is clearly one of the most important stimuli in our auditory environment, which not only conveys speech information, but allows us to recognize individuals and their emotional states (Belin et al., 2004). In human adults, voices are processed in specialized brain regions located in the upper bank of the superior temporal sulcus (Belin et al., 2000). Recently, it has been shown that macaque monkeys have a similar voice-selective region in the superior temporal plane that preferentially responds to conspecific vocalizations, suggesting that recognizing the sound that is the vocalization of a species member is an evolutionarily conserved brain function in primates that is independent of language (Petkov et al., 2008, 2009). These voice-selective areas in auditory cortex, similar to face-selective areas in visual cortex identified in both human adults and monkeys (Kanwisher et al., 1997; Tsao et al., 2006), are thought to bind the processing of crucial socially relevant information to sensory systems.

In human adults, the voice-sensitive temporal regions not only react to voice-specific information but are moreover sensitive to emotional prosody crucial in social communication (Grandjean et al., 2005; Ethofer et al., 2006). Such a modulation of sensory processing by emotional signals is particularly strong for threat-related emotions, occurs independent of attention, and is thought to be a fundamental neural mechanism both in face- and voice-sensitive brain regions to prioritize the processing of significant stimuli (see Vuilleumier, 2006, for a review). Although well described for the adult brain, the developmental origins of the cortical organization underlying voice and emotional prosody processing in the human brain remain unknown. Here we report two experiments with young infants that fill this gap.

Behavioral work has shown that newborn infants prefer human voices to similar nonsocial auditory stimuli (Ecklund-Flores and Turkewitz, 1996; Hutt et al., 1968) and their mother's voice to the voice of another newborn's mother (DeCasper and Fifer, 1980). These postnatal listening preferences are primarily related to infants' sensitivity to prosodic characteristics of speech (Mehler et al., 1988; Moon et al., 1993). The latter finding is relevant insofar as prosodic cues are known to play an essential role in the perception of vocally communicated emotions (Scherer, 1986). Indeed, newborns of English- and Spanish-speaking mothers presented with a range of vocal expressions (happy, angry, sad, and neutral) in their native and nonnative language showed an increase in eye-opening responses following the onset of stimuli with happy prosody when compared to the other emotions, but only when they listened to the vocal expression in their native language (Mastropieri and Turkewitz, 1999). Despite this very early form of sensitivity to happy prosody in familiar contexts, further behavioral studies show that only from around 5 months of age do infants robustly discriminate between happy, angry, and sad emotional prosody (Flom and Bahrick, 2007; Walker-Andrews, 1997).

Recent electrophysiological work indicates an early sensitivity to language-specific and emotion-specific prosodic information in the speech signal. The processing of prosodic stress was shown to elicit language-specific event-related brain potentials (ERPs) in 4- to 5-month-old infants (Friederici et al., 2007). An ERP study investigating the processing of emotional prosody in 7-month-old infants (Grossmann et al., 2005) revealed that infants discriminated between neutral, happy, and angry emotional prosody. As early as 300 ms poststimulus onset, ERPs for angry prosody differed from happy or neutral prosody over frontal and central electrodes, suggesting a greater initial attention to angry voices. Both angry and happy prosody resulted in a greater positive slow wave than neutral prosody at temporal electrodes, pointing toward an enhanced sensory processing of emotionally loaded stimuli. Thus it appears that aspects of the human voice and prosody, be it emotional or intonational, are processed early in life and that the brain reacts quite specifically to these aspects in speech (for reviews of auditory language functions during early infancy, see Friederici, 2006; Kuhl, 2004).

Although this work has provided important insights, ERP data cannot provide clear information on the exact brain regions that are involved in processing prosody in infancy. Studies investigating the brain substrates of infants' auditory discrimination abilities by measuring their hemodynamic brain responses indicate that, already by the age of 2 months, infants display a left hemispheric advantage for spoken language, whereas music results in bilateral patterns of activation in the planum temporale (Dehaene-Lambertz et al., 2009). Furthermore, a right hemispheric advantage for the processing of language prosody in the temporal cortex can be observed by the age of 3 months (Homae et al., 2006). These lateralization patterns are quite similar to those seen in adults (for reviews see Vigneau et al., 2006; Friederici and Alter, 2004; Koelsch and Siebel, 2005). However, despite the similar brain lateralization patterns, 2- to 3-month-old infants do not yet show specificity in their brain responses in temporal cortex. Namely, direct contrasts between speech and music, mother's and stranger's voice (Dehaene-Lambertz et al., 2009), and forward and backward speech (Dehaene-Lambertz et al., 2002) did not reveal significant differences in 2- to 3-month-olds' temporal cortex responses. This suggests that the specialization of temporal brain regions involved in speech and voice recognition occur after the age of 3 months.

The present study used near-infrared spectroscopy (NIRS) permitting spatial localization of brain activation by measuring hemodynamic responses to investigate the neurotopography of voice and emotional prosody in young infants (see Minagawa-Kawai et al., 2008; Lloyd-Fox et al., 2010 for reviews of this method and its use with infants). Other neuroimaging techniques that are well established in adults are limited in their use with infants because of methodological concerns. For example, positron emission tomography (PET) exposes participants to radioisotopes, and functional magnetic resonance imaging (fMRI) requires the participant to remain very still and exposes them to a noisy environment. Although both PET and fMRI have been used with infants, this work is restricted to the study of sleeping, sedated, or very young infants. NIRS is better suited for infant research because it can accommodate a good degree of movement from the infants, enabling them to sit upright on their parent's lap and behave relatively freely while watching or listening to certain stimuli. In addition, unlike PET and fMRI, NIRS systems are portable. Finally, despite its inferior spatial resolution, NIRS, like fMRI, measures localized patterns of hemodynamic responses, thus allowing for a comparison of infant NIRS data with adult fMRI data (see Strangman et al., 2002, for evidence of a strong correlation between the hemodynamic responses measured with fMRI and NIRS).

We first investigated voice sensitivity in infants, as voices have been shown to be processed in specific temporal brain regions in human adults and nonhuman primates (Petkov et al., 2008, 2009). In experiment 1, we thus presented 4- and 7-month-old infants with vocal and nonvocal sounds, in order to examine when regions in infant temporal cortices become sensitive to the human voice. We decided to study infants of these ages as prior work suggests that speech and specific voices (e.g., mother's voice) do not yet evoke adult-like specialized temporal brain responses in younger infants (Dehaene-Lambertz et al., 2009). Second, we assessed whether the voice-sensitive regions as identified in experiment 1 were modulated by emotional prosody (Grandjean et al., 2005; Ethofer et al., 2006). In experiment 2, we therefore presented 7-month-old infants with happy, angry, and neutral prosody while measuring their brain responses.

## Results

### Experiment 1

**Figure 1:**
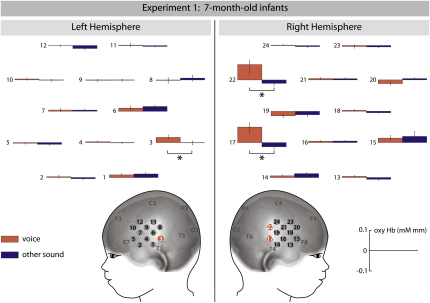
Voice-Sensitive Brain Regions Identified in 7-Month-Old Infants in Experiment 1 This graph depicts mean oxygenated hemoglobin concentration changes (±SEM) for vocal and other sounds measured from 24 NIRS channels. Channels that showed significant increases for vocal compared to other sounds are marked in red on the head model.

**Figure 2:**
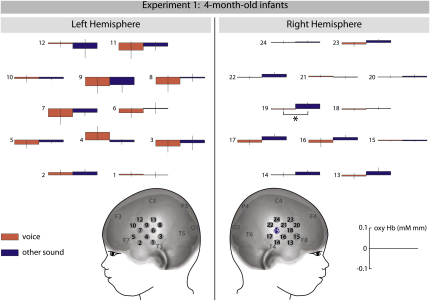
Brain Responses in 4-Month-Old Infants in Experiment 1 This graph depicts mean oxygenated hemoglobin concentration changes (±SEM) for vocal and other sounds measured from 24 NIRS channels. The channel that showed a significant increase for other sounds compared to vocal sounds is marked in blue on the head model.

Our analysis of 7-month-old infants' brain responses revealed that three channels in posterior temporal cortex, two located in the right hemisphere (channel 17 and 22) and one located in the left hemisphere (channel 3), were sensitive to the human voice (see Figure 1). These three brain regions showed significant increases in oxygenated hemoglobin (oxyHb) concentration when the vocal condition was compared to the nonvocal condition (left hemisphere: channel 3: F [1, 15] = 4.782, p = 0.045; right hemisphere: channel 17: F [1, 15] = 5.626, p = 0.032 and channel 22: F [1, 15] = 5.797, p = 0.029). Similar increased activation effects were not obtained in our analysis of 4-month-old infants' brain responses (see Figure 2). Rather, there was one region in the right hemisphere that showed significant increases in oxyHb when the nonvocal condition was compared to the vocal condition (channel 19: F [1, 15] = 5.07, p = 0.04). For the group of 7-month-olds, no brain regions were found in which the oxyHb concentration changes were higher in the nonvocal than in the vocal condition.

The analysis of deoxygenated hemoglobin (deoxyHb) concentration changes revealed no significant differences between conditions in 4- and 7-month-old infants. The fact that we did not find any significant decreases in deoxyHb that accompanied the increase in oxyHb, as one would expect on the basis of adult work (Obrig and Villringer, 2003), is in line with previous infant NIRS work (Grossmann et al., 2008; Meek, 2002; Nakato et al., 2009). Several infant NIRS studies either failed to find a significant decrease or even observed an increase in deoxyHb concentration. Although a number of factors such as immaturity of the infant brain have been suggested to explain this difference between infants and adults, the exact nature of this difference remains an open question (for a discussion, see Meek, 2002; Nakato et al., 2009).

### Experiment 2

**Figure 3:**
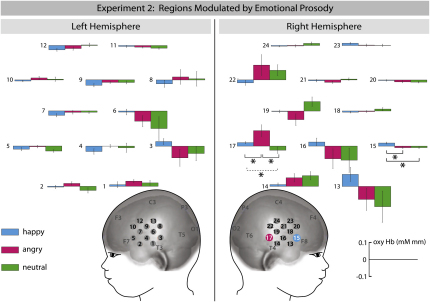
Brain Regions Modulated by Emotional Prosody in Experiment 2 This graph depicts mean oxygenated hemoglobin concentration changes (±SEM) for happy, angry, and neutral prosody measured from 24 NIRS channels. The channel that showed an increased sensitivity to angry prosody is marked in magenta, and the channel that showed increased sensitivity to happy prosody is marked in blue on the head model.

Our analysis revealed two channels in the right hemisphere (channels 15 and 17) that were sensitive to emotion in 7-month-old infants (see Figure 3). These channels showed significant differences in oxyHb concentration when emotion (happy, angry, and neutral prosody) was assessed as a within-subjects factor in repeated-measures ANOVAs (right hemisphere: channel 15: F [2, 34] = 7.245, p = 0.002; channel 17: F [2, 34] = 4.977, p = 0.013). Of these two channels, channel 17 (located in posterior temporal cortex) had been identified as voice sensitive in experiment 1. This channel showed a significant increase in oxyHb when the angry condition was compared to the happy condition (t [1, 17] = 2.165, p = 0.045) and when the angry condition was compared to the neutral condition (t [1, 17] = 2.289, p = 0.035) using a post-hoc paired t test. Furthermore, channel 17 also showed an increase in oxyHb that was marginally significant when the happy condition was compared to the neutral condition (t [1, 17] = 2.052, p = 0.056). Moreover, channel 15, located in the right inferior frontal cortex, showed a significant increase in oxyHb when the happy condition was compared to the angry condition (t [1, 17] = 2.943, p = 0.009) and when the happy condition was compared to the neutral condition (t [1, 17] = 2.765, p = 0.013), whereas the angry condition was not statistically different from the neutral condition (t [1, 17] = 0.102, p = 0.92). As in experiment 1, the analysis of deoxy concentration changes revealed no significant differences between conditions.

## Discussion

The present study investigated the processing of voice specificity and prosody specificity in the infant brain.

### Voice Processing

In experiment 1, we found that 7-month-old infants showed significantly increased hemodynamic responses in left and right superior temporal cortex to the human voice when compared to nonvocal sounds. This suggests that voices, as a class of auditory objects with high occurrence and ecological interest, are processed in a fairly specialized brain region by 7 months of age. Strikingly, 4-month-old infants' temporal regions did not show a similar voice-sensitive responding in experiment 1, indicating that voice sensitivity in the posterior temporal cortex emerges between 4 and 7 months of age. The finding that the group of younger infants did not show voice-sensitive responding is in line with earlier fMRI work in which 2- to 3-month-olds failed to show adult-like increased temporal cortex responses when speech was compared to backward speech (Dehaene-Lambertz et al., 2002) or music (Dehaene-Lambertz et al., 2009). Infants by the age of 4 months rather showed an increased hemodynamic response to nonvocal stimuli in one region in right temporal cortex located more anterior then the region identified as voice sensitive in 7-month-olds. This finding suggests that 4-month-olds' brains are able to discriminate between the two kinds of auditory stimuli but they seem to be using different (immature) brain mechanisms for this discrimination, since only 7-month-olds show adult-like increased responses to the human voice.

The brain region identified as voice sensitive in 7-month-olds appears to be localized in similar portions of the superior temporal cortex as in adults (see Belin et al., 2000, and Figure S1 for comparison of localization in adults), indicating developmental continuity in voice processing between 7-month-old infants and adults. In adults, the voice-sensitive regions for stimulus material identical to that used in the present experiment 1 were found in the upper bank of the superior temporal sulcus (Belin et al., 2000). However, the spatial precision in localizing cortical responses achieved with NIRS in infants is more coarse than the excellent spatial resolution obtained by fMRI used in previous adult studies (see Aslin and Mehler, 2005; Lloyd-Fox et al., 2010 for a discussion of the advantages and limitations of using NIRS with infants). Furthermore, our current measurement technique did not provide us with information of the depth at which the source of this activation is located (see Blasi et al., 2007, for NIRS methodology that allows for the measurement of depth-dependent hemodynamic responses in infants). Therefore, we cannot assess whether the voice-sensitive regions identified in 7-month-old infants are located in the sulcus or the gyrus of the superior temporal cortex. Nevertheless, the functionally similar brain responses in superior temporal cortex in infants and adults suggest that the current infant NIRS results and previous fMRI results with adults represent homologous brain processes. Taken together, in conjunction with earlier work with nonhuman primates (Petkov et al., 2008), by demonstrating that this brain specialization emerges early during human postnatal development, the results of experiment 1 provide further support for the notion that sensitive responding to the vocalizations of conspecifics is an evolutionarily important brain function in primates.

### Processing Emotional Prosody

The brain responses to emotional prosody as obtained in experiment 2 are in line with previous adult studies (Grandjean et al., 2005; Ethofer et al., 2006). Hearing emotional prosody (happy and angry) but not neutral prosody evoked an increased response in a right temporal region in 7-month-old infants that was identified as voice sensitive in experiment 1. This result indicates that the enhancement of sensory processing by emotional signals is a fundamental and early developing neural mechanism engaged to prioritize the processing of significant stimuli (Vuilleumier, 2006). It is interesting to note that the brain response in right temporal cortex was larger to angry prosody when compared to happy prosody, indicating that threatening signals have a particularly strong impact on voice processing (see also Grossmann et al., 2005). This heightened sensitivity to negative information is in accordance with the notion of a negativity bias, which is proposed to be an evolutionarily driven propensity to attend and react more strongly to negative information (Cacioppo and Berntson, 1999) that appears to emerge in the second half of the first year of life (see Vaish et al., 2008).

In experiment 2, hearing happy prosody but not angry or neutral prosody, evoked an increased response in a region in right inferior frontal cortex in 7-month-olds that did not show voice sensitivity in experiment 1. Greater activation to happy voices than angry voices in right inferior frontal cortex has also been observed in adults (Johnstone et al., 2006), suggesting developmental continuity in how the human brain processes happy prosody. Current models of prosody processing in adults (Schirmer and Kotz, 2006; Wiethoff et al., 2008) hold that, following the acoustic analysis in temporal cortices, information is passed on to the inferior frontal regions for further and more detailed evaluation. The finding that 7-month-olds engage right inferior frontal cortex when listening to happy prosody might therefore indicate that speech characterized by positive vocal affect undergoes a more explicit evaluation than speech with neutral or angry affect.

This finding might also relate to a number of behavioral findings suggesting that infants show strong perferences for infant-directed speech (so-called motherese). Motherese compared to adult-directed speech posseses unique acoustic characteristics: it is generally slower and contains exaggerated pitch contours, hyperarticulation of vowels, and (critical for the interpretation of the current findings) positive prosody (Fernald, 1985; Kuhl et al., 1997; Cooper and Aslin, 1990). It is also interesting to note that motherese with its happy prosody has been found to facilitate learning, specifically language and word learning in the developing infant (Kuhl, 2004; Liu et al., 2003; Singh et al., 2002; Vallabha et al., 2007). Therefore, in conjunction with these behavioral findings, the inferior frontal response to happy prosody observed in 7-month-old infants in experiment 2 may constitute the neural basis for a more detailed cognitive evaluation of infant-directed happy speech.

### Role of the Right Hemisphere

Even though voice-sensitive responses were observed in both hemispheres in 7-month-olds in experiment 1, the right hemisphere seemed to be more interested in voice compared to other sounds. While only one NIRS channel showed a voice-sensitive response in the left hemisphere, two adjacent voice-sensitive channels were found in the right hemisphere. Moreover, the overall magnitude of the responses to voices in the two channels in the right hemisphere was larger than that in the left hemisphere. The finding that the spatial extent and the magnitude of the voice-sensitive response were larger in the right hemisphere is in line with adult imaging findings suggesting that the voice-sensitive responses are predominant in the right hemisphere (Belin et al., 2000). The modulation of infant brain responses by emotion observed in experiment 2 was restricted to the right hemisphere. Similarly, in adult neuroimaging studies, responses in temporal cortex showed strongest effects of emotion in the right hemisphere (Grandjean et al., 2005; Ethofer et al., 2006). In conjunction with some lesion work (Borod et al., 2002), this has led to the suggestion that the right hemisphere plays a predominant role in processing emotional prosody (Wildgruber et al., 2002). However, in adults, lesion studies have also discussed the contribution of the left hemisphere for the understanding of emotional prosody (Kucharska-Pietura et al., 2003; Ross et al., 1997; Van Lancker and Sidtis, 1992). But this can be explained by the fact that in these adult lesion studies meaningful speech stimuli were used, and the left hemisphere is thought to be involved in the recognition of emotion conveyed through meaningful speech (Kucharska-Pietura et al., 2003). The right hemisphere shows a clear dominance for prosodic information once any lexical information is absent in the acoustic stimuli (for a review, see Friederici and Alter, 2004). The current data from 7-month-old infants together with those from adults suggest that voice-sensitive regions in the right hemisphere play an important role in processing emotional prosody.

### Implications for Neurodevelopmental Disorders

Finally, these findings might also have important implications for neurodevelopmental disorders such as autism. Adult participants with autism fail to activate voice-sensitive regions in temporal cortex (Gervais et al., 2004). Furthermore, older children and adults with autism are impaired in identifying emotion expressed through tone of voice (Hobson et al., 1989; Rutherford et al., 2002; Van Lancker et al., 1989). Our findings demonstrating that voice-sensitive brain regions are already specialized and modulated by emotional information by the age of 7 months raise the possibility that the critical neurodevelopmental processes underlying impaired voice processing in autism might occur before 7 months. Therefore, in future work the current approach could be used to assess individual differences in infants' responses to voices and emotional prosody and might thus serve as one of potentially multiple markers that can help with an early identification of infants at risk for a neurodevelopmental disorder (for example, see Elsabbagh and Johnson, 2007).

## Experimental Procedures

### Participants

The final sample in experiment 1 consisted of 16 7-month-old infants (eight girls) aged between 201 and 217 days (M = 210.2 days) and 16 4-month-old infants (seven girls) aged between 108 and 135 days (M = 123.1 days). The final sample in experiment 2 consisted of 18 7-month-old infants (eight girls) aged between 199 and 216 days (M = 211.8 days). An additional 26 were tested for experiment 1 (4 months: n = 6; 7 months: n = 8) and experiment 2 (7 months: n = 12) but not included in the final sample because they had too many motion artifacts resulting in too few usable trials for analysis (minimum number of five trials per condition) (n = 18) or because of technical failure (n = 2). Note that an attrition rate at this level is within the normal range for an infant NIRS study (Minagawa-Kawai et al., 2008; Lloyd-Fox et al., 2010). All infants were born full-term (37–42 weeks gestation) and with normal birthweight (>2500 g). All parents gave informed consent before the study.

### Stimuli

For experiment 1, stimulus material consisted of 40 8 s long trials of vocal and nonvocal sounds (16 bit/22 KHz sampling rate). Vocal trials included speech (words and nonwords) as well as nonspeech vocalizations, and nonvocal trials consisted of sounds from nature, animals, modern human environment (cars, telephone, airplanes), and musical instruments (for more detail, see Belin et al. [2000] and http://vnl.psy.gla.ac.uk). For experiment 2, the stimulus material consisted of 74 semantically neutral German verbs previously validated and used with adults (Schirmer and Kotz, 2006) and with infants (Grossmann et al., 2005). A female speaker produced all words with happy, angry, and neutral prosody. Words were taped with a DAT recorder and digitized at a 16 bit/44.1 kHz sampling rate. The three emotions did not differ with respect to their mean intensity (for further acoustic analysis, see Grossmann et al., 2005).

### Procedures

Infants were seated on their parent's lap in a dimly lit and sound-attenuated room. Stimuli were presented via loudspeaker (SPL = 70 dB). In experiment 1, the experimental sessions consisted of 8 s long trials during which various vocal or nonvocal sound stimuli were presented consecutively. Voices and nonvocal sounds were randomly distributed over the session with no more than two trials of the same category occurring in a row. The intertrial interval was 12 s. In experiment 2, the experimental session consisted of 5 s long trials during which five words of one emotion category (happy, angry, or neutral) were presented consecutively. Trials from the different emotional categories were randomly distributed over the session with no more than two trials of the same category occurring consecutively. The intertrial interval was 15 s. During the presentation of the acoustic stimuli, a cartoon was presented to the infants on a computer screen placed at a 60 cm distance in order to keep their attention and reduce motion artifacts. The experimental session lasted on average 7 min, 20 s (average number of trials = 22).

Data acquisition and analysis. In both experiments, cortical activation was measured using a Hitachi ETG-4000 NIRS system. The multichannel system uses two wavelengths at 695 nm and 830 nm. Two custom-built arrays consisting of nine optodes (five sources, four detectors) in a 12 channel (source-detector pairs) arrangement with an interoptode separation of 20 mm were placed over temporal and inferior frontal brain regions on each hemisphere (see Figures 1–3) using an Easycap (Falk Minow). The NIRS method relies on the optical determination of changes in oxygenated (oxyHb) and deoxygenated (deoxyHb) hemoglobin concentrations in cerebral cortex, which result from increased regional cerebral blood flow (Obrig and Villringer, 2003). NIRS data were continuously sampled at 10 Hz. For analysis, after calculation of the hemoglobin concentration changes, pulse-related signal changes and overall trends were eliminated by low-pass filtering (Butterworth, 5^th^ order, lower cutoff 0.5 Hz). Movement artifacts were corrected by an established procedure (see Koch et al., 2006; Wartenburger et al., 2007), which allows marking of artifacts and then padding the contaminated data segments by linear interpolation. Cortical activations were assessed statistically by comparing average concentration changes (oxyHb and deoxyHb) within trials (20 s after stimulus onset) between the experimental conditions by using repeated-measures ANOVAs.
