# Supplementary material for: The Developmental Origins of Voice Processing in the Human Brain
Source: Neuron. 2010 Mar 25;65(6):852–8. doi: 10.1016/j.neuron.2010.03.001 (PMC2852650; doi:10.1016/j.neuron.2010.03.001)

Neuron, Volume 65 Supplemental Information

The Developmental Origins of Voice Processing in the Human Brain

Tobias Grossmann, Regine Oberecker, Stefan Paul Koch, and Angela D. Friederici

Figure S1. Voice-sensitive regions in the adult human brain, related to Figure1. This figure (adapted from Petkov, Logothetis, & Obleser, 2009) provides the exact localization of voice-sensitive regions in adults (Belin et al., 2000) and presents them in reference to the electrode positions T3 and T4, thus, allowing for a comparison with the infant findings that are also presented with reference to these electrode positions.

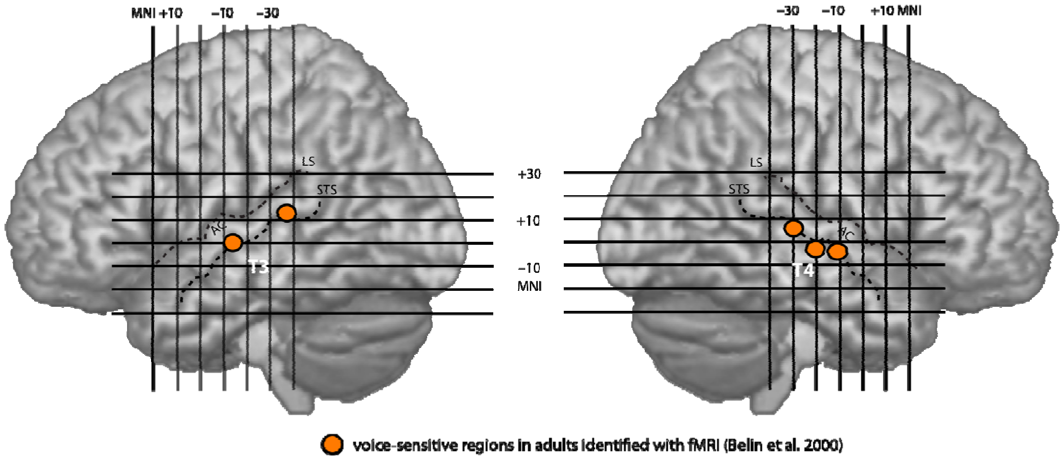

Supplement: Document S1. Supplemental Figure [file mmc1.pdf]
